# Multifactorial day hospital intervention to reduce falls in high risk older people in primary care: a multi-centre randomised controlled trial [ISRCTN46584556]

**DOI:** 10.1186/1745-6215-7-5

**Published:** 2006-02-27

**Authors:** Tahir Masud, Carol Coupland, Avril Drummond, John Gladman, Denise Kendrick, Tracey Sach, Rowan Harwood, Pradeep Kumar, Rob Morris, Rachael Taylor, Jane Youde, Simon Conroy

**Affiliations:** 1 Clinical Gerontology and Research Unit, Nottingham City Hospital, Hucknall Road, Nottingham, NG5 1PB, UK; 2 School of Community Health Sciences, University of Nottingham, Nottingham, NG7 2UH, UK; 3 Department of Health Care for the Elderly, Queen's Medical Centre, Nottingham, NG7 2UH, UK; 4 Department of Health Care for the Elderly, Derbyshire Royal Infirmary, London Road, Derby, DE1 2QY, UK

## Abstract

Falls in older people are a major public health concern in terms of morbidity, mortality and cost. Previous studies suggest that multifactorial interventions can reduce falls, and many geriatric day hospitals are now offering falls intervention programmes. However, no studies have investigated whether these programmes, based in the day hospital are effective, nor whether they can be successfully applied to high-risk older people screened in primary care.

The hypothesis is that a multidisciplinary falls assessment and intervention at Day hospitals can reduce the incidence of falls in older people identified within primary care as being at high risk of falling. This will be tested by a pragmatic parallel-group randomised controlled trial in which the participants, identified as at high risk of falling, will be randomised into either the intervention Day hospital arm or to a control (current practice) arm. Those participants preferring not to enter the full randomised study will be offered the opportunity to complete brief diaries only at monthly intervals. This data will be used to validate the screening questionnaire. Three day hospitals (2 Nottingham, 1 Derby) will provide the interventions, and the University of Nottingham's Departments of Primary Care, the Division of Rehabilitation and Ageing Unit, and the Trent Institute for Health Service Research will provide the methodological and statistical expertise. Four hundred subjects will be randomised into the two arms. The primary outcome measure will be the rate of falls over one year. Secondary outcome measures will include the proportion of people experiencing at least one fall, the proportion of people experiencing recurrent falls (>1), injuries, fear of falling, quality of life, institutionalisation rates, and use of health services. Cost-effectiveness analyses will be performed to inform health commissioners about resource allocation issues. The importance of this trial is that the results may be applicable to any UK day hospital setting.

**Sites:**

General practices across Nottinghamshire and Derbyshire.

Day hospitals:

Derbyshire Royal Infirmary (Southern Derbyshire Acute Hospitals NHS Trust)

Sherwood Day Service (Nottingham City Hospital Trust)

Leengate Day Hospital (Queen's Medical Centre Nottingham University Hospital NHS Trust)

## Background

Falls are a common and serious problem facing elderly people and are associated with considerable mortality and morbidity [1]. Forty to sixty percent of falls cause injuries with an estimated 5% of incident falls resulting in fractures[2,3]. Falls result in institutionalisation[4], hospitalisation[5] and injury-related death[6]. Over the age of 65 years a third of the population fall at least once a year [7] rising to over a half by 85 years [2]. Previous studies have identified a number of potentially modifiable fall risk factors[1]. Intervention studies in the USA and New Zealand have shown that a combined multidisciplinary assessment and treatment programme and an individualised home based targeted exercise programme respectively can reduce falls by 30%-46% [8-10]. The UK PROFET study showed that in patients presenting to an Accident and Emergency department with a fall, the number of subsequent falls were reduced by half in those who had received the intervention, which constituted a thorough medical assessment and an occupational therapy home visit[11].

In 1999 there were 647,721 A&E attendances and 204,424 admissions to hospitals in the UK as a result of injuries sustained in unintentional falls by the over 60's. It was calculated that these falls cost the UK Government a total of £981 million, £581 million of which was met by the NHS. The main cost components were inpatient admissions (making up 49.4% of total costs) and long term care costs (accounting for 41%). The costs incurred as the result of a fall increased with age[12].

We do not know whether interventions provided to high risk older people identified in primary care are effective, nor whether falls programmes now offered by day hospitals are sufficiently well constituted and intensive to deliver similar benefits to those studies outlined above. Nor do we know if day hospital settings will be acceptable to the 'screened well'. Addressing 'fear of falling' and consequent social isolation or restriction are key features of an effective falls prevention programme. The proposed multi-centre randomised controlled trial will assess whether falls can be successfully and cost-effectively reduced using day hospital facilities.

In addition to assessing the effectiveness of the Day hospital as an intervention to reduce falls it is important to evaluate the intervention in terms of its cost-effectiveness. In a resource constrained health care system it is important to ensure that limited resources are spent in such a way as to maximise outcomes. Within the context of this proposed study the economic objective is to establish whether it is efficient to allocate resources to the Day hospital, in comparison to standard care, as an intervention to reduce falls amongst elderly people. To address the efficiency of putting resources to Day hospitals a cost-effectiveness analysis and cost-utility analysis will be performed.

## Objectives

### Main research hypothesis and question

The main hypothesis to be tested is that a multidisciplinary falls assessment and intervention occurring at a Geriatric Day hospital can decrease the rate of falls over the course of one year, in older people identified in primary care as being at high risk of falling.

### Secondary research questions

1. Can the above intervention reduce the proportion of people with single or recurrent falls (>1)?

2. Can the above intervention reduce fall-related injuries (including fractures)?

3. Can the above intervention reduce disability and improve quality of life?

4. Can the above intervention reduce institutionalisation and the need for the use of health services?

5. Is the intervention cost-effective and might it lead to overall cost-savings?

6. Can a screening questionnaire used in primary care reliably distinguish between low and high risk of falling?

7. Is there any difference in deaths between the two groups?

### Subject definition

The study population will comprise men and women aged 70 and over identified at being at high risk of falling by a postal screening questionnaire, registered with the participating general practices in Nottinghamshire and Derbyshire.

### Exclusion criteria

Patients already attending one of the day hospitals

Patients under follow-up with an existing primary care based falls prevention scheme or day hospital falls service

Residents in nursing or residential homes

Patients with terminal illnesses

Those unwilling or unable to travel to the day hospital (using transport as provided)

## Study design and treatment definition

### Study design

The proposed study is a pragmatic parallel group randomised controlled trial where the participants will be randomised into either the intervention day hospital arm or to a control (current practice) arm. After outcome measurement has taken place those in the control arm will be offered the day hospital intervention.

### Intervention & control groups

The two arms of the study are as follows:

• Intervention arm: screening questionnaire, information leaflet, leaflet on falls prevention and invitation to attend the day hospital for assessment and any subsequent intervention

• Control arm: screening questionnaire, information leaflet, leaflet on falls prevention and usual care from primary care service until outcome data collected, then offer of day hospital intervention.

## Procedures and observations

In most cases, subjects will be contacted by post and telephone at each stage of the study; however, it is likely that some individuals will be unable or unwilling to use these methods in which case they will be offered a home visit.

### Baseline measurements

Participating general practices will be asked to identify all patients aged 70 and over in their practice. Practices will be asked to exclude all those living in a nursing or residential home and those with terminal illness. The remaining subjects will be sent an invitation to participate by their general practice.

The invitation will include the patient information leaflet as well as a screening questionnaire which the subjects will be asked to return to the project officer. This screening questionnaire is based on published guidelines[1] and will be adapted for the local population in a pilot study. Those subjects not participating in the randomised study will be offered the opportunity to complete the monthly diaries only; data from these replies will be used to explore the predictive strengths of the screening questionnaire. The screening questionnaire will enable the identification of subjects deemed to be at high risk of falling and will include questions on previous falls history, mobility, use of walking aids and the number of medications. In addition to returning the questionnaire, subjects will be asked if they would be interested in participating in the study and if so to provide contact details, including phone number. The screening questionnaire will also ask about other exclusion criteria such as attendance at the day hospital. The replies will be analysed by the project officer and suitable participants for the study identified. Potential participants will be asked if they have had contact with either the day hospital or the primary care falls prevention service in the preceding year; in case of any doubt, verbal consent will be obtained to check their possible contact with these services with the day hospital or the primary care team.

A written invitation will be sent offering a home visit from the study nurse where no telephone contact is possible.

Those eligible subjects thought to be at high risk of future falls (approximately 1000) will then be sent a pack containing further information on the study, a falls prevention booklet ('Avoiding slips, trips and broken hips', Department of Health) and a consent pack.

The subjects will then be contacted by phone with the aim of:

• Clarifying any issues about the study

• Confirm responses to the screening questionnaire

• Invitation to take part

• Obtaining verbal consent (based on a standardised schema) and requesting signing and return of consent form in prepaid envelope

On receipt of the signed consent form, the project officer will then contact the randomisation centre. Subjects will be informed of the next steps by phone, with confirmation by post within 48 hours. This second letter will include information on which arm they have been randomised, the diaries for recording falls and if appropriate, an appointment for the day hospital.

For those willing to take part but who do not want to be part of the main RCT, there will be the option of returning a brief falls diary and the end of study questionnaire only.

### Outcome measurements

#### Definition of primary and secondary outcomes

Primary outcome: rate of falling over the 12 month follow-up period.

Falls will be recorded by giving each participant a diary and reply paid envelopes. The definition of a fall given to subjects in this study will be 'inadvertently coming to rest on the ground or other lower level with or without loss of consciousness and other than as a consequence of sudden onset of paralysis, epileptic seizure, excess alcohol intake, or overwhelming external force' (PROFET[11]).

Participants will be asked to record falls in the diary, along with the outcome (saw GP, phoned ambulance, sent to hospital, injuries). The diaries will be mailed back to the research team at the end of every month. Participants will be contacted via telephone by the "blinded" assessor at the end of each month to encourage return of the diary. Falls will be monitored until, withdrawal from study, death or end of 12 month follow up, which ever event occurs first.

Secondary outcomes:

1. Proportion of people with single or recurrent falls (>1)... defined as above

2. Fall-related injuries: fracture, serious sprain requiring immobilisation in plaster, joint dislocations, head injury requiring hospitalisation, and lacerations requiring suturing

3. Disability: Nottingham Extended Activities of Daily Living Scale; Barthel index of daily living; Quality of life: Falls Efficacy Scale and EuroQoL-5

4. Institutionalisation and use of health services: residency and diary information

5. Cost analysis

6. Screening tool... defined by sensitivity/specificity as well as positive and negative predictive values

7. Deaths will be checked against PCT records and measured as proportions

#### Ascertainment of outcomes

The principal method will be self-reporting using the diary. This will contain a section on falls and the outcomes (carried on as usual, called for help, waited for someone to help, called GP, called ambulance, taken to hospital, nature of any injury). Additionally, there will be a section recording health service utilisation, covering primary care contacts and hospital admissions.

The self-reported data will be cross referenced where possible through access to GP records and hospital information systems. For hospital admissions, access to the case notes will be requested and the notes reviewed. This level of information will allow a more accurate ascertainment of events as well as the costs associated. Information on falls-related drug treatment (such as bone protection therapy) will be obtained from the GP records. These data will be collected by the project manager and project officer on standardised forms. The data collection will be subject to internal audit to ensure accurate and consistent data recording and avoid intraobserver and interobserver bias.

Institutionalisation data will be available from practice registers. Those who change practice as a result of changing address will be traced through the primary care trusts.

Disability and quality of life will be self reported by study participants. They will be asked to complete the Nottingham extended activities of daily living scale (NEADL)[24], Barthel index of Daily Living, EuroQol[25] and the falls efficacy scale[26]. These will be posted at the end of one year, along with reply paid envelopes. Non-responders will be sent a written reminder and if necessary a follow-up phone call ('hot pursuit').

EuoQol is included to facilitate cost-effectiveness and cost-utility calculations, but the Falls Efficacy Scale (FES) is a more sensitive measure of quality of life in this population, hence its inclusion[27]. The FES has been validated in the UK as both a face to face and self completed questionnaire[27]. The intraclass correlation coefficient is 0.58, suggesting fair agreement when used as a postal questionnaire. Both the Barthel index and the NEADL are well validated measures of functional ability and can be used as a postal questionnaire[24].

#### Patients not recruited into the study

Data will be available from the screening questionnaire for those patients who prefer not to participate in the randomised trial or who are excluded as per the study protocol, but who agree to complete the monthly diaries and end of study booklet. Using this data it will be possible to check the representative nature of the study population and the discriminatory power of the screening questionnaire. Those subjects who prefer not to take part in the study at either level will not be contacted any further. They will have access to usual care through their GPs/primary care teams.

### Randomisation

After giving informed consent patients will be randomly allocated (1:1 ratio) to either the intervention group or to the control group. Randomisation will be made by telephone to the TRDSU who will be blind to the identity of the patient, and will use a computer program (Stata) to carry out stratified block randomisation based on study centre (Nottingham, Derby).

### Treatment

#### Intervention arm

The intervention arm will be invited to attend the day hospital for an assessment and any subsequent intervention, including:

##### Medical assessment and treatment performed by a clinician

History (including medications) [standard format]; a full physical examination including visual acuity and orthostatic blood pressure measurement; laboratory tests where indicated; 12 lead ECG and where appropriate a neurovascular assessment (carotid sinus massage and tilt tests). Treatments will vary according to the medical diagnoses made and will include medication review, appropriate referral to an optician or ophthalmologist for visual impairment and referral to other specialists where necessary.

##### Physiotherapy assessment and individualised therapy programme conducted by a physiotherapist

Assessment for gait, balance, mobility and muscle strength (using the Tinetti[15] method and measurement of ankle dorsiflexion strength[16]). Interventions will include gait re-education and functional training programme using the principles of Koch[17], and muscle strengthening exercise programme based on a modified Dunedin protocol[10], and where indicated the provision of an appropriate walking aid. The feet and footwear will be assessed for abnormalities that could impair gait and appropriate referrals will be made to a chiropodist or an orthotist. The number and timings of follow up visits at the day hospital for further assessment and therapy and telephone calls to check compliance with the programme will depend on the individualised needs of the participants.

##### Occupational therapy assessment and modifications, performed by an occupational therapist

Interview with the participant using a standardised checklist to investigate home hazards[18]. Where necessary, a home visit will then also be performed. Participant's functioning will be assessed using a standard activities of daily living measure[19]. Modification of identified environmental hazards will be suggested according to published criteria[18].

#### Control arm

In addition to the falls prevention leaflet, the control group will receive all routine care provided by their primary care service. This may or may not include referral to secondary care, depending on the GP's judgement. At present, there are primary care falls prevention services being developed, but in an inconsistent manner. Participation in the control arm may include referral to such services as part of normal practice, but these referrals will not constitute part of the trial protocol. Patients in the intervention arm will be asked not to participate in the primary care falls prevention services for the duration of the trial. Assuming the trial does not show a detrimental effect from the day hospital intervention, the control group will be offered day hospital attendance at the end of the trial.

### Contamination

Pilot data from the Nottingham Community Osteoporosis (NOCOS) study[20] suggest that very few older patients identified as fallers are referred to secondary care or other agencies for a falls assessment (personal communication with Trial Co-ordinator). Although contamination between groups is a potential limitation, our previous experience suggests this is unlikely to be an important issue. We will specifically check to see if any of the control group have attended secondary care services for a falls evaluation. Currently all referrals to the primary care falls prevention services are centralised within each primary care trust; through checking with these centres, we will be able to ascertain who in the control group received a primary care based intervention. We will also use this point of contact to ensure that those in the day hospital arm of the trial are not also receiving a community intervention or follow-up.

### Withdrawal

Participants wishing to withdraw from the study will be free to do so at any stage, without any detriment to their usual care.

### Concurrent treatment

There are no other falls prevention studies running in this area at present that might be relevant. Community falls prevention programmes are being developed, but we will liaise with these groups to ensure that the day hospital group are not put into these services. The control group will be able to access these services in the usual fashion.

### Data analysis

#### Power and sample size

With an expected rate of falls of 2/year per person[11], and an over dispersion parameter of 1.5 a clinically important risk reduction of 24% to 1.5 falls/year can be detected with 80% power and 5% significance (two-sided) in a trial of 160 participants in each arm giving a trial size of 320. This assumes a Poisson distribution with over dispersion. If the fall rate was 1/year per person[10] a risk reduction of 33% could be detected with this sample size (not a high risk population). To allow for an attrition rate of 20% (see below) we will recruit a total of 400 participants. To achieve this, 3125 subjects will need to be screened, of whom 40% may be at high risk of falls. Of these 1250, 20% are likely to be ineligible leaving 1000 potential participants of whom it is thought that 40% will agree to take part. This sample size will also have 80% power at the 5% significance level to detect a reduction of one third in the proportion of people with one or more falls, with an expected proportion with one or more falls of 50% at 12 months.

#### Data monitoring and analysis

Data will be double entered into Access to check data entry validity and analysis performed using SPSS (version 11) and Stata (version 8). Intention to treat analysis will be used. Analysis will performed according to the pre-specified analysis plan.

#### Univariate and multivariate analyses

For the primary analysis, Poisson regression or if there is over-dispersion (likely), negative binomial regression will be used to estimate the incidence rate ratio comparing the rate of falls between the two randomised groups during the follow-up period. The number of falls will be the numerator and length of follow-up the denominator.

Secondary analysis will compare the following outcomes between treatment arms:

a) The proportion of people with one or more falls over the follow-up period, using logistic regression

b) Time to first fall using survival analysis

c) the proportion of people with injurious falls using logistic regression

d) the proportion of people institutionalised at 12 months using logistic regression

e) Impact on disability, quality of life, fear of falls, and use of health services will be assessed using linear regression models with transformations if necessary, although some of these comparisons will be underpowered.

f) Deaths will be compared between the two groups. The analysis will be survival analysis using Cox proportional hazards model.

All analyses will adjust for the strata used in the randomisation procedure. If there are other important baseline differences, for example in age or number of previous falls, multivariate analysis will be used to adjust for them.

#### Subgroup analyses

We will perform subgroup analysis for age (70–85, 85+) and falls history in the last year (0–1, ≥2). The statistical significance of differences between subgroups will be tested using tests for interaction in the regression models.

#### Economic analyses

Cost-effectiveness analysis taking a societal perspective, including NHS, patient and social services support will be carried out using established methods[28]. Incremental cost-effectiveness ratios will be calculated based on the duration of the trial only.

Resource use data will be collected with respect to inpatient and outpatient staff time (Consultants, GP, nurses, physiotherapists, and occupational therapists), Overhead costs, diagnostic tests (radiology and blood services), dressings, medication, transport costs (NHS and private), patient out-of-pocket costs and patient/carer time costs. Resource use data will be collected using a report form abstracting from patient records as well as the patient diaries at monthly intervals throughout the trial for both the intervention and control groups. These data will be cross referenced, where appropriate, with GP records and prescribing systems.

Health and social care resource use data will be valued using published unit cost data[22,23] Social care items will include the number and duration of carer visits per day, and the use of home food delivery services. Time costs will be valued using published wage rates from the new earnings survey and patient out-of-pocket costs will be reported directly by the patient.

The principal outcome measure will be the number of falls prevented to give the incremental cost per fall prevented and Quality Adjusted Life Years as measured on the EuroQol EQ-5D to produce the cost per QALY.

No discounting will be necessary as the trial period is 12 months. One way sensitivity analysis of extremes to test the robustness of the estimated incremental cost-effectiveness ratios will be undertaken.

### Project management and administration

#### Project management

The project will be managed day to day by the project manager (SC) working in close partnership with the project officer (RT) and study secretary (Stephanie Sutcliffe). A steering group will meet monthly (SC, RT, TM, DK) and the overall study group will meet three-monthly (all investigators).

The project will first start in Nottingham; RT and Dr Drummond will be responsible for the training and preparation of the day hospital staff as well as the preparation of the participating practices. The project will then be rolled out sequentially to Derby, with RT and SC providing close support.

We will be seeking to ensure that the core intervention is uniform across the day hospitals, although some local variation is to be expected. The uniformity of the intervention will be monitored using audit and direct observation (initially weekly, later monthly and then three monthly – AD). The day hospitals will be assessed to ensure that:

• the local set up and structure is appropriate

• the appropriate staff are providing the appropriate interventions

The financial management of the project will be controlled by SC and TM, with quarterly reviews with the investigators

#### Procedure for recruiting general practices

The collaborative research network (CRN) is an established network of General Practices in Trent who have indicated their willingness to be involved in studies. Subject to their approval of this study, we will use this network to recruit willing practices.

The day hospitals in Derby and Nottingham, along with their respective physicians have been recruited already.

#### Trial documentation

The documents required to be completed and by whom are highlighted in table 1.

**Table 1:** Trial documentation

**Form title**	**Completed/sent by**
Screening questionnaire	Potential participant
Invitation pack (PIL, falls advice, consent)	Potential participant
No phone letter v1	Project officer
Consent form	Potential participant
Confirmatory letter (randomised group, diary 1, DH appointment)	Project officer
Exclusion letter to pts	Project officer
Diary completion reminder	Project officer
Diary 2–11	Participant
Acute hospital event form/case note abstraction	Project officer
GP event form/case note abstraction	Project officer
Day hospital resource use	Multidisciplinary team
Final outcome pack (FES, NEADL, EuroQoL, diary 12)	Potential participant
Follow-up only falls enquiry	Non-RCT follow up only participant

#### Record retention

Data will be stored in compliance with data protection act. All records will be stored for seven years from the date of the last study publication

#### Payments

We are unable to offer any payments to participants, however, transport to and from the day hospital will be provided, along with meals and refreshments. The general practices will be each paid £60 in lieu of their time and effort in disseminating the original screening questionnaire and affording subsequent access to records. The project officer and manager will be reimbursed for any relevant travel expense. Attendance at the group meetings by the investigators will also be reimbursed.

#### Project milestones

See table 2.

**Table 2:** Project milestones

**From**	**To**	**Activity**
4/2004	8/04	Planning & preparation. Procurement of honorary contracts
6/2004		Submission to COREC and Research & Development offices
8/04	12/04	Pilot phase (Nottingham). Preparation of other participating centres
12/04		Start of recruitment – Nottingham.
3/05		First interim analysis (Nottingham)
1/06		First centre – one year results
4/07		Final data analysis
4/07	8/07	Writing of report and papers for publication, preparing dissemination through presentation at local, national and international meetings, preparation for applications for further peer-reviewed grants

### Informed consent

Verbal consent will be obtained from all participants where possible. Participants will be asked to return a signed consent form indicating that they have received sufficient information during the telephone conversation. They will have the option of being visited at home if the telephone medium hinders fully informed verbal consent.

In the event of communication difficulties, the project officer will be able to visit the participant and their advocate in their home with a view to obtaining written consent or assent from the advocate. There will be no formal testing of cognitive function, but if it is felt that the potential participant will not be able to follow the required study protocol and/or attendances at the day hospital, then they will be excluded.

The study website will be made available to participants and their relatives, giving further information on falls prevention[29].

### Confidentiality

All participant questionnaires and case note abstraction forms will be stored in locked cabinets, identified by a unique participant identifier. Consent forms and other documents including the participants name will be stored separately from questionnaires and other trial documents in locked cabinets. All computer databases will include the unique participant identifier and not the name and address of the participant.

### Publications

All investigators will contribute towards drafting the paper reporting the main trial findings and all investigators will be named authors on that paper, providing they fulfill the Vancouver criteria for authorship.

Investigators wishing to analyse and report other findings from the study can do so on the agreement of the other investigators, and the study team will agree authorship for these papers, subject to the Vancouver criteria for authorship.

It has been agreed by the study team and project officer that SC (project manager) will be able to use the study and the data for the basis of his PhD thesis.

## Authors' contributions

• TM and RH developed the original idea, study design and obtained pump-priming funding. SC contributed to the study design and obtinaed the main funding. CC, AD, JG, DK, PK, RM, TS and JY all contributed to study design and are pump-priming grant holders. RT improved on the study design and helped with data collection. All authors read and approved the final manuscript. The authors declare that they have no competing interests.

## Ethics approval

COREC approval has been obtained from the Nottingham main REC and the relevant local RECs (reference: 04/Q2404/93).

Chief investigator: Pr Masud; principal investigators at other sites:

◦ Dr P Kumar (Queen's Medical Centre)

◦ Dr J Youde (Derby Royal Infirmary)

The primary care trusts will be designated as 'no local investigator' sites.

Trial sponsor: Nottingham City Hospital NHS Trust

**Figure 1:**
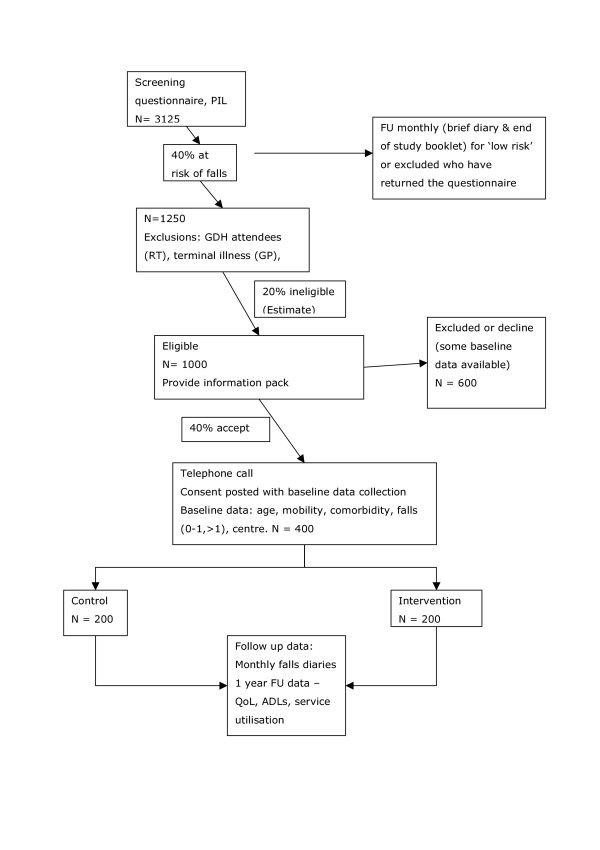
Participant flow.
